# COVID-19 Vaccine Knowledge, Practice, and Attitudes Among Hemodialysis Patients in Egypt, Kenya, and Cameroon: A Multicenter Study

**DOI:** 10.1016/j.xkme.2026.101465

**Published:** 2026-07-14

**Authors:** Enass Elsayed, Khaled M. Kotb, Ahmed Heiba, Manal Shaker Elhussini, Ahmed Abdelmoniem Emara, Nzana Victorine Bandolo, Hala Mahfouz Abd Elmageed Khalil, Basma Arif Eltaweel, Marwa Shaban Abdel Samea, Shereen Omar Ahmed, Radwa Ibrahim, Eman E.A. Mohammed, Alaa Abdelhamid Ali, Khalida B. Soki, Mohammed Abdel Gawad

**Affiliations:** 1 Brigham and Women's Hospital, Boston, MA; 2 Harvard Medical School, Boston, MA; 3 Department of Nephrology, Ahmad Maher Teaching Hospital, GOTHI, Cairo, Egypt; 4 Clinical Pharmacy and Pharmacy Practice Department, Faculty of Pharmacy, Mansoura University, Mansoura, Egypt; 5 Department of Environmental and Occupational Health, School of Public Health, Texas A&M University, College Station, TX; 6 Internal Medicine Department, National Research Centre, Dokki, Cairo, Egypt; 7 Gastroenterology and Infectious Diseases Department, Ahmed Maher Teaching Hospital, Cairo, Egypt; 8 Pulmonology Department, Ahmed Maher Teaching Hospital, Cairo, Egypt; 9 Nephrology Department, Ain Shams University, Cairo, Egypt; 10 Department of Internal Medicine and Specialties, Faculty of Medicine and Biomedical Sciences, University of Yaounde I, Yaounde, Cameroon; 11 Internal Medicine Department, Ahmed Maher Teaching Hospital; 12 Nephrology Department, National Institute of Urology and Nephrology, Egypt; 13 Nephrology Department, Ain Shams University, Cairo, Egypt; 14 Nephrology Department, Electricity Hospital, Cairo, Egypt; 15 Department of Internal Medicine–Nephrology Unit, Assiut University Hospital, Faculty of Medicine, Assiut University, Assiut 71711, Egypt; 16 Medical Molecular Genetics Department, Human Genetics and Genome Research Institute, National Research Centre, Cairo, Egypt; 17 Department of Nephrology, Ahmed Maher Teaching Hospital, GOTHI, Cairo, Egypt; 18 Nephrology Department, The Nairobi Hospital, Nairobi, Kenya; 19 Nephrology Unit, Internal Medicine Department, School of Medicine, Newgiza University (NGU), Giza, Egypt

**Keywords:** Hemodialysis, COVID-19, vaccination, vaccine hesitancy, knowledge, attitudes

## Abstract

**Rationale & Objective:**

Patients receiving hemodialysis (HD) are at increased risk of severe coronavirus disease 2019 (COVID-19) and were prioritized for vaccination, yet vaccine hesitancy remains common. We assessed COVID-19 vaccine knowledge, acceptance, and attitudes among patients receiving HD in Egypt, Kenya, and Cameroon and identified factors associated with vaccine acceptance, prior infection, willingness to receive future doses, and postvaccination complications.

**Study Design:**

Multicenter cross-sectional survey study.

**Setting & Participants:**

Between March 2021 and April 2022, 765 patients receiving maintenance HD and 196 non-dialysis controls were recruited from dialysis centers in Egypt, Kenya, and Cameroon.

**Predictor(s):**

Sociodemographic characteristics, clinical comorbidities, prior COVID-19, sources of vaccine information, and exposure to vaccinated or infected relatives.

**Outcome(s):**

COVID-19 vaccine acceptance, willingness to receive future doses, prior infection, and post-vaccination complications.

**Analytical Approach:**

Structured questionnaires assessed knowledge, practices, and attitudes toward COVID-19 vaccination. Multivariable logistic regression identified factors independently associated with study outcomes.

**Results:**

Vaccine acceptance was lower among patients receiving HD than nondialysis controls (58.2% vs 96.2%). Fear of side effects was the most common reason for refusal (39.3%). Postvaccination complications were less frequent among patients receiving HD (22.3% vs 44.3%). Hesitancy was more common among women, younger participants, and those with comorbidities or no prior COVID-19. Having vaccinated or previously infected relatives was associated with a greater willingness to receive future doses.

**Limitations:**

Cross-sectional design limits causal inference. Nonprobability sampling and unmatched controls may limit generalizability. COVID-19 cases may have been underreported because of limited testing.

**Conclusions:**

COVID-19 vaccine hesitancy among patients receiving HD is driven by fear, misinformation, and sociodemographic factors. Targeted education and improved access to reliable vaccine information may help increase uptake in this population.

## Plain Language Summary

Patients receiving hemodialysis are at high risk of severe coronavirus disease 2019 (COVID-19), but little is known about their attitudes toward vaccination. We conducted a study across multiple hospitals in Egypt, Kenya, and Cameroon to understand how these patients and a control group of nondialysis individuals perceive COVID-19 vaccines. Participants were asked about their knowledge, previous vaccination, willingness to receive future doses, and any side effects experienced. We found that most patients accepted vaccination, although some hesitated because of concerns about side effects. These findings highlighted factors that influence vaccine decisions in vulnerable populations and provide important insights for health care providers and policymakers. Understanding these patterns can help design strategies to increase vaccine uptake and protect patients receiving dialysis.

## Introduction

Patients receiving hemodialysis (HD) are susceptible to coronavirus disease 2019 (COVID-19) infection because of multiple comorbidities and dialysis units’ close-contact environment.^1^ Furthermore, logistical aspects of HD may further increase the risk of disease transmission.^2^, ^3^, ^4^ The nephrology community issued guidance to mitigate infection risks among this vulnerable population.^5^, ^6^, ^7^, ^8^

By early 2021, at least 7 COVID-19 vaccines had been introduced worldwide, and the COVAX initiative was launched to ensure equitable vaccine access for vulnerable populations. Despite these efforts, vaccine hesitancy emerged as a major global public health concern.^6^

In Egypt, vaccines such as AstraZeneca, Sinopharm, Sinovac, Sputnik V, and Johnson & Johnson were deployed, and local production of Sinovac began.^7^ However, surveys revealed that nearly half of Egyptians (48%) were uncertain about receiving vaccination, and data on vaccine awareness or attitudes among patients receiving HD were lacking.^6^

In Cameroon, overall vaccine acceptance in the general population was low—only about one-third expressed willingness to be vaccinated—driven by fear of side effects, misinformation, and limited trust in authorities.^8^ These contextual factors suggest similar challenges may exist among patients receiving HD in both countries.

Given these gaps, this study aimed to assess the knowledge, attitudes, and practices related to COVID-19 vaccination among maintenance patients receiving HD in Egypt, Kenya, and Cameroon, while also evaluating prevaccination infection rates and post-vaccination complications.

## Methods

### Study Design and Population

**Figure 1:**
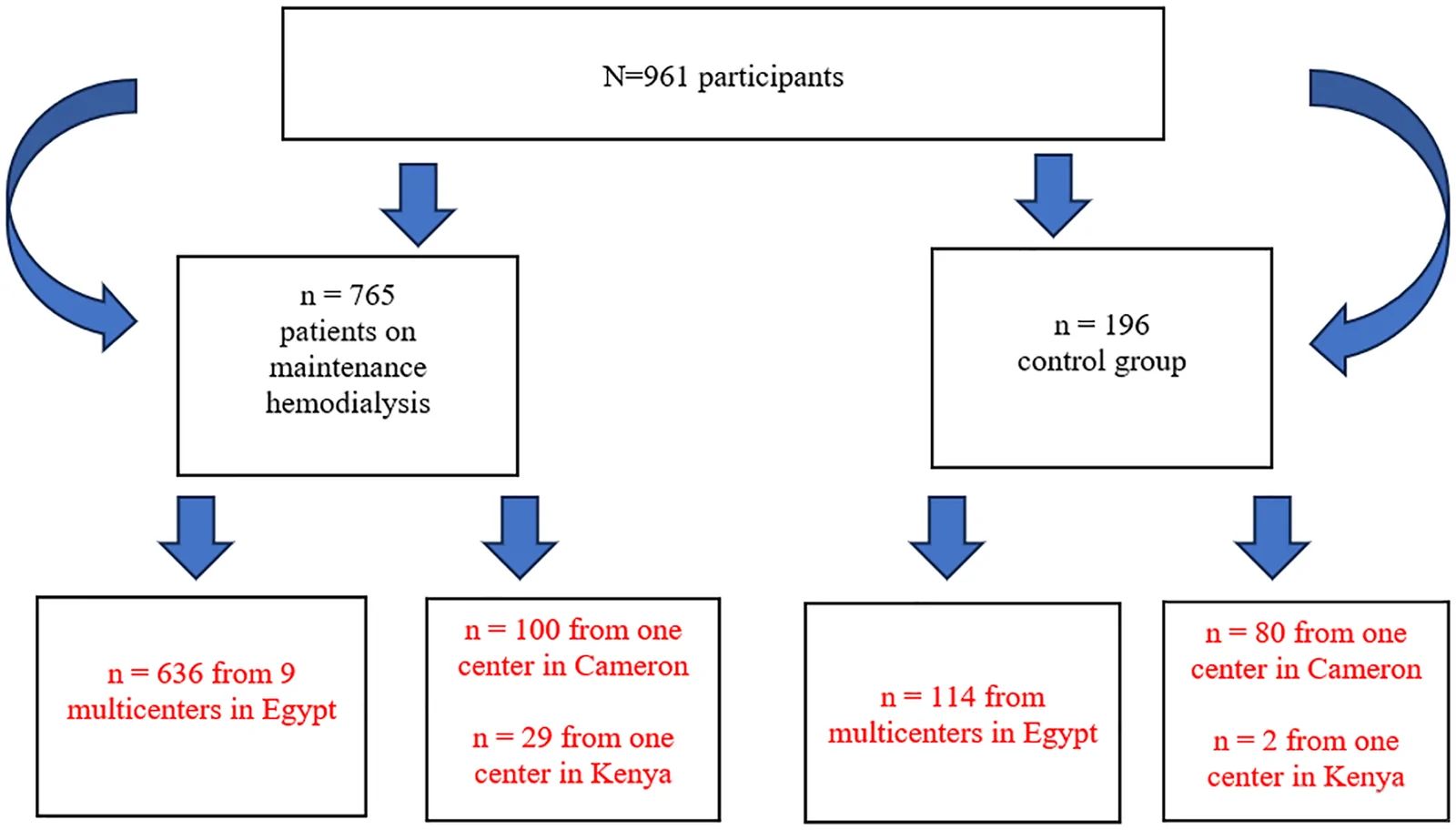
Recruitment flowchart of study participants.

This multicenter cross-sectional survey study included a total of 765 patients receiving HD, recruited from 11 HD units with 636 patients from Egypt, 100 from Cameroon, and 29 from Kenya. and 196 were nondialysis controls recruited from patients' relatives from 2021 to 2022 (Fig 1). Participants were enrolled consecutively. Differences in participant numbers across centres reflect variation in patient volumes and response rates.

All participating dialysis centers followed standard infection control measures during the study period, including mandatory mask-wearing, hand hygiene, temperature screening, and patient education regarding COVID-19 precautions.

The study was approved by the local institutional review boards of all participating centers. Written informed consent was obtained from all participants before enrollment. All procedures were conducted in accordance with the Declaration of Helsinki.

Nondialysis controls were adults aged ≥18 years recruited from relatives of HD patients at participating centers. Controls were not formally matched to the HD group because of logistical constraints, which may limit generalizability.

The inclusion criteria were adults aged 18 years or older with kidney failure receiving maintenance HD and their nondialysis relatives as controls. Patients unwilling to participate, aged younger than 18 years, and those with acute kidney injury or earlier stages of chronic kidney disease were excluded.

The study aimed to assess the knowledge of patients receiving HD regarding COVID-19 vaccination by identifying their main sources of information; evaluate their attitudes through the analysis of demographic, clinical, and COVID-19-related factors associated with vaccination acceptance; and examine their practices by assessing their intention to receive future vaccine doses. Additionally, the study sought to determine the rate of COVID-19 before vaccination and to describe postvaccination complications.

Data were collected using structured questionnaires administered by nephrologists at the participating hospitals (Supplementary Item S1). The questionnaire covered 5 main sections: sociodemographic characteristics, clinical history (including the cause of renal failure [Figure S1]), laboratory data, COVID-19 history, and attitudes toward COVID-19 vaccination. Domains associated with vaccine hesitancy—including perception of disease severity, sources of disease and vaccine information, and confidence in the healthcare system—were also explored.

### Exposure Variables

Predictors were categorized into 3 domains: demographic, COVID-19 perception, and clinical factors. Demographic predictors included age, sex, marital status, working status, smoking status, and HD duration. COVID-19 perception predictors encompassed previous history of COVID-19, COVID-19 reinfection, relatives infected with COVID-19, relatives who died from COVID-19, relatives who received the COVID-19 vaccine, and patients who received influenza vaccination. Clinical predictors were the presence of comorbid conditions (diabetes, hypertension, cardiac disease, and anemia^9^), complications during HD (hypertension, hypotension, muscle cramps). Model discrimination and multicollinearity were assessed using the receiver operating characteristic area under the curve and variance inflation factors.

### Outcome Variables

Primary outcomes were vaccination status and willingness to receive the future dose. The secondary outcomes were COVID-19 before vaccination and post-vaccination complications.

### Statistical Analyses

Data distribution was examined graphically; normally distributed data were represented as mean (± standard deviation), while median (interquartile range) was used to represent nonnormally distributed variables. Frequencies and corresponding proportions were used to represent categorical variables. The *t* test, χ^2^ test, and the nonparametric tests were used to compare groups as appropriate for the data distribution.

Multivariable logistic regression analyses were conducted to identify factors associated with COVID-19 before vaccination, vaccination status, willingness to receive the future dose, and postvaccination complications among patients receiving HD.

Three separate models were constructed: model 1 (demographic), model 2 (COVID-19 perception), and model 3 (clinical), using the predictor domains described above.

Variables included in the multivariable logistic regression were selected based on clinical relevance and prior literature, not solely on univariate significance. This approach allows adjustment for potential confounders and prevents omission of clinically important variables. Additionally, marital status was included as a socio-demographic variable potentially influencing health behaviors, including vaccine uptake.

Model discrimination and multicollinearity were assessed using the receiver operating characteristic area under the curve and variance inflation factors.

Statistical significance was set at a 2-tailed *P* value < 0.05. All analyses were conducted using the STATA 17 statistical package (Stata Corp).

## Results

### Baseline characteristics

#### Patients Receiving HD and the Control Group

Data were available for (n=765) patients receiving HD and (n=196) nondialysis participants as controls, from 11 centers in Egypt, Kenya, and Cameroon (Fig 1). At baseline, patients receiving HD were significantly older ( 49.9 ± 15) than nondialysis controls (36.1 ± 10.73), and 29.7% of them were older than 60 years versus 3.3% of controls, with a predominance of males (56.8%). A significantly lower proportion of patients receiving HD were highly educated (43.9%) compared with controls (97.6%), with fewer employed (44.8%) compared with controls (92.7%). History of COVID-19 before vaccination was significantly less common in patients receiving HD (22.6%) than in controls (53.2%). COVID-19 vaccination was significantly lower in patients receiving HD (58.2%) compared with controls (96.2%). Among those who received the vaccine, willingness to receive future doses was significantly lower in patients receiving HD (73.8%) versus controls (78.0%). Postvaccination complications in those who received the vaccine were significantly less common in patients in the HD group (22.3%) than in controls (44.3%) (Table 1).

**Table 1:** Baseline Characteristics Comparing Patients Receiving Maintenance Hemodialysis, and Nondialysis Controls

Factor	Level	N	Patients receiving HD Total=765	N	Control Total=196	*P-*value
Nationality, n (%)	Egyptian	765	636 (83.10)	196	114 (58.20)	< 0.001
Age, y, mean ± SD		743	49.94 ± 15.14	184	36.10 ± 10.73	< 0.001
Age >60 y, n (%)		743	221 (29.70)	184	6 (3.26)	< 0.001
Sex, n (%)	Male	764	434 (56.80)	190	95 (50.00)	0.09
Educational level, n (%)	Illiterate	314	75 (23.90)	82	0 (0.00)	< 0.001
	Low		101 (32.20)		2 (2.40)	
	High		138 (43.90)		80 (97.60)	
Employment, n (%)	Employed	328	147 (44.80)	82	76 (92.70)	
	Not working		98 (29.90)		0 (0.00)	
	Manual worker		58 (17.70)		6 (7.30)	
	Retired		25 (7.60)		0 (0.00)	
Currently working, n (%)			205 (62.50)		82 (100.00)	< 0.001
Smoking satus, n (%)	Current	679	100 (14.70)	165	5 (3.00)	< 0.001
	Previous		87 (12.80)		4 (2.40)	
	Never		492 (72.50)		156 (94.50)	
Diabetes, n (%)		765	40 (5.23)	196	0 (0.00)	0.001
Hypertension, n (%)			323 (42.22)		0 (0.00)	< 0.001
Cardiac disease, n (%)			18 (2.35)		0 (0.00)	< 0.001
Anemia, n (%)			422 (55.16)		16 (8.16)	< 0.001
Any comorbidity, n (%)			526 (68.76)		16 (8.16)	
History of COVID-19, n (%)		641	145 (22.60)	124	66 (53.20)	< 0.001
Received COVID-19 treatment (%)			50 (34.48)		3 (4.55)	< 0.001
No. of reinfections, n (%)	1	133	124 (85.52)	66	54 (81.82)	0.008
	2		6 (4.14)		9 (13.64)	
	3		2 (1.38)		3 (4.55)	
	4		1 (0.69)		0 (0.00)	
Relatives had COVID-19, n (%)		736	52 (7.10)	194	28 (14.40)	0.001
Relatives died of COVID-19, n (%)		541	70 (12.90)	194	35 (18.00)	0.08
Relatives vaccinated, n (%)		735	117 (15.90)	194	35 (18.00)	0.48
Hemoglobin level, g/dL, mean ± SD		432	9.61 ± 1.74	27	12.07 ± 1.98	< 0.001
Influenza vaccinated, n (%)		525	39 (7.42)	114	7 (6.14)	0.63
COVID-19 vaccinated, n (%)		637	371 (58.20)	130	125 (96.20)	< 0.001
Postvaccine complications, n (%)		371	164 (44.2)	125	86 (68.8)	< 0.001
Willing for future doses, n (%)		301	222 (73.75)	109	85 (77.98)	< 0.001
No. of vaccine doses, n (%)	1	280	163 (58.21)	87	37 (41.40)	0.01
	2		113 (40.36)		46 (52.87)	
	3		4 (1.43)		4 (4.60)	

Results are presented as proportions, n (%) for categorical variables and mean ± standard deviation for normally distributed continuous variables.

### Predictors of COVID-19 Before Vaccination

In total, 22.6% of patients receiving HD had COVID-19 before vaccination. In the univariate analysis, older age (model 1) was significantly associated with marginally increased odds of COVID-19 (odds ratio [OR], 1.01; 95% confidence interval [CI], 1.001-1.03). Participants who had anemia (model 2) were more likely to have had a COVID-19 (OR, 3.10; 95% CI, 2.06-4.67). Having relatives who received the COVID-19 vaccine (model 3) was associated with lower odds of infection (OR, 0.49; 95% CI, 0.28-0.86). In multivariable analysis, participants who had anemia (model 2) were more likely to have had a COVID-19 (OR, 5.74; 95% CI, 3.47-9.47; Table 2).

**Table 2:** Predictors of COVID-19 Before Vaccination and Postvaccination Complications Among Patients Receiving Hemodialysis

Predictors	COVID-19		Postvaccination complications	
	OR (95% CI) Univariate analysis	OR (95% CI) Multivariable analysis	OR (95% CI) Univariate analysis	OR (95% CI) Multivariable analysis
Model 1: Demographic predictors				
Age, y	**1.01 (1.001-1.03)**	1.00 (0.98-1.03)	1.00 (0.99-1.01)	0.99 (0.96-1.02)
Sex				
Female	0.95 (0.65-1.38)	1.15 (0.49-2.71)	1.17 (0.82-1.65)	1.80 (0.59-5.50)
Marital status				
Married	1.10 (0.74-1.64)	1.08 (0.51-2.29)	**2.51 (1.63-3.86)**	**5.83 (2.00-16.97)**
Working status				
Working	1.57 (0.79-3.11)	1.88 (0.86-4.07)	0.59 (0.27-1.27)	0.98 (0.36-2.68)
Smoking				
Current	0.61 (0.35-1.09)	0.79 (0.22-2.80)	1.24 (0.74-2.08)	**6.14 (1.40-26.88)**
Previous	0.84 (0.47-1.51)	0.91 (0.31-2.66)	1.32 (0.75-2.31)	2.35 (0.49-11.30)
Hemodialysis duration (mo)	1.00 (0.99-1.00)	1.00 (0.99-1.00)	1.00 (1.00-1.00)	**1.01 (1.006-1.02)**
Model 2: Comorbidities predictors				
Diabetes comorbidity	1.66 (0.73-3.76)	1.72 (0.61-4.86)	1.00 (omitted)	1.00 (omitted)
Hypertension comorbidity	1.18 (0.80-1.72)	2.05 (0.93-4.52)	**0.28 (0.19-0.42)**	1.00 (omitted)
Cardiac disease comorbidity	0.98 (0.32-3.01)	0.96 (0.27-3.40)	1.00 (omitted)	1.00 (omitted)
Anemia	**3.10 (2.06-4.67)**	**5.74 (3.47-9.47)**	1.10 (0.77-1.57)	1.07 (0.66-1.73)
Any comorbidities^a^	**1.91 (1.24-2.94)**	—	**0.52 (0.36-0.75)**	—
Had hypertension during HD	0.82 (0.41-1.65)	0.37 (0.12-1.08)	1.22 (0.63-2.35)	2.20 (0.70-6.92)
Had hypotension during HD	0.72 (0.39-1.36)	0.40 (0.15-1.09)	**2.27 (1.34-3.85)**	2.32 (0.83-6.46)
Model 3: COVID-19-related predictors				
History of COVID-19	—	—	0.87 (0.56-1.34)	**22.65 (5.24-97.91)**
Reinfection^a^	—	—	**0.45 (0.30-0.68)**	0.04 (0.01-0.16)
Relatives had COVID-19	1.45 (0.76-2.79)	1.81 (0.74-4.41)	1.46 (0.78-2.72)	1.31 (0.57-3.03)
Relatives died with COVID-19	0.75 (0.39-1.44)	0.78 (0.22-2.80)	**2.68 (1.57-4.60)**	2.51 (0.45-14.15)
Relatives received the vaccine	**0.49 (0.28-0.86)**	0.60 (0.19-1.89)	**2.75 (1.80-4.19)**	0.44 (0.08-2.44)
Influenza vaccine	0.71 (0.33-1.51)	0.82 (0.27-2.51)	1.47 (0.80-2.68)	0.82 (0.27-2.51)

Bold values indicate statistically significant differences.

Abbreviations: CI, confidence interval; OD, odds ratio.

a Excluded from the final model due to multicollinearity concerns, as it overlaps conceptually and statistically with individual comorbidity variables included in the model.

### Factors Associated With COVID-19 Vaccination Acceptance

Among patients receiving HD, 58.2% were found to be vaccinated. In univariate analysis in model 1, older age (OR, 1.02; 95% CI, 1.01-1.03), being married (OR, 1.94; 95% CI, 1.39-2.72), and HD duration (OR, 1.004; 95% CI, 1.001-1.01) were associated with increased odds of vaccination, whereas female gender was associated with lower odds of vaccination (OR, 0.70; 95% CI, 0.51-0.96). In model 2, diabetes (OR, 0.43; 95% CI, 0.19-0.97), hypertension (OR, 0.36; 95% CI, 0.26-0.50), and cardiac disease (OR, 0.27; 95% CI, 0.09-0.76) and muscle cramps during HD (OR, 0.48; 95% CI, 0.30-0.78) were inversely associated with vaccination, as were any HD complications (OR, 0.49; 95% CI, 0.34-0.70). In model 3, having relatives who received the COVID-19 vaccine was strongly associated with higher vaccine acceptance (OR, 2.88; 95% CI, 1.80-4.61). Interestingly, COVID-19 reinfection was associated with decreased odds of being vaccinated (OR, 0.69; 95% CI, 0.50-0.96).

In the multivariable analysis, married participants (model 1) (OR, 2.52; 95% CI, 1.29-4.94) with hypertension comorbidity (OR, 0.34; 95% CI, 0.18-0.66) (model 2), history of COVID-19 (OR, 2.54; 95% CI, 1.41-4.57), and COVID-19 reinfection (OR, 0.35; 95% CI, 0.21-0.59) (model 3) were more likely to accept the vaccine (Table 3).

**Table 3:** Predictors of Vaccination Acceptance and Willingness to Receive Next Doses Among Patients Receiving Hemodialysis

Predictors		Vaccination acceptance		Receiving the next dose	
		OR (95% CI) Univariate analysis	OR (95% CI) Multivariable analysis	OR (95% CI) Univariate analysis	OR (95% C I) Multivariable analysis
Model 1: Demographic predictors					
Age, y		**1.02 (1.01-1.03)**	1.01 (0.98-1.04)	1.01 (0.99-1.02)	1.00 (0.98-1.03)
Sex	Female	**0.70 (0.51-0.96)**	1.44 (0.69-3.00)	0.97 (0.65-1.44)	1.07 (0.50-2.31)
Marital status	Married	**1.94 (1.39-2.72)**	**2.52 (1.29-4.94)**	1.44 (0.96-2.18)	1.40 (0.69-2.83)
Working status	Working	1.49 (0.84-2.63)	1.88 (0.94-3.77)	1.21 (0.65-2.26)	1.13 (0.55-2.33)
Smoking	Current	1.23 (0.78-1.93)	1.97 (0.66-5.83)	1.05 (0.59-1.84)	2.09 (0.69-6.38)
	Previous	**1.74 (1.03-2.91)**	1.03 (0.41-2.52)	0.92 (0.51-1.65)	0.88 (0.35-2.27)
Hemodialysis duration (mo)	—	**1.004 (1.001-1.01)**	1.003 (1.00-1.01)	1.00 (0.996-1.002)	1.00 (0.995-1.005)
Model 2: Comorbidities predictors					
Diabetes comorbidity	—	**0.43 (0.19-0.97)**	0.76 (0.30-1.94)	**0.34 (0.14-0.83)**	0.63 (0.23-1.73)
Hypertension comorbidity	—	**0.36 (0.26-0.50)**	**0.34 (0.18-0.66)**	**0.52 (0.34-0.78)**	**0.38 (0.18-0.79)**
Cardiac disease comorbidity	—	**0.27 (0.09-0.76)**	0.37 (0.12-1.13)	**0.25 (0.09-0.71)**	**0.31 (0.10-0.97)**
Anemia	—	0.81 (0.59-1.11)	1.15 (0.74-1.80)	0.96 (0.65-1.43)	0.76 (0.45-1.26)
Any comorbidities^a^	—	**0.48 (0.34-0.69)**	—	**0.49 (0.31-0.77)**	-
Had hypertension during HD	—	0.77 (0.44-1.37)	1.49 (0.57-3.89)	**0.51 (0.26-0.98)**	0.68 (0.19-2.37)
Had hypotension during HD	—	1.45 (0.85-2.49)	1.78 (0.73-4.35)	1.20 (0.67-2.15)	0.93 (0.29-3.01)
Had muscle cramps during HD	—	**0.48 (0.30-0.78)**	1.40 (0.55-3.56)	0.89 (0.52-1.51)	1.31 (0.39-4.41)
Any HD complication	—	**0.49 (0.34-0.70)**	0.77 (0.34-1.75)	1.20 (0.67-2.15)	1.39 (0.45-4.31)
Model 3: COVID-19-related predictors					
History of COVID-19	—	0.81 (0.55-1.18)	**2.54 (1.41-4.57)**	0.89 (0.52-1.51)	1.66 (0.86-3.20)
Reinfection	—	**0.69 (0.50-0.96)**	**0.35 (0.21-0.59)**	0.77 (0.52-1.15)	0.56 (0.32-0.97)
Relatives had COVID-19	—	1.77 (0.91-3.46)	1.33 (0.54-3.32)	2.90 (1.06-7.88)	1.97 (0.54-7.16)
Relatives died with COVID-19.	—	1.32 (0.75-2.32)	0.40 (0.14-1.17)	1.80 (0.86-3.76)	**0.06 (0.01-0.51)**
Relatives received the vaccine	—	**2.88 (1.80-4.61)**	2.37 (0.92-6.06)	**2.38 (1.38-4.08)**	**33.80 (4.15-275.39)**
Influenza vaccine		2.23 (0.96-5.16)	1.24 (0.47-3.27)	1.93 (0.88-4.28)	2.11 (0.59-7.48)

Bold values indicate statistically significant differences.

Abbreviations: CI, confidence interval; OD, odds ratio.

a Excluded from the final model because of multicollinearity concerns because it overlaps conceptually and statistically with individual comorbidity variables included in the model.

### Reasons for Not Receiving the COVID-19 Vaccine

**Figure 2:**
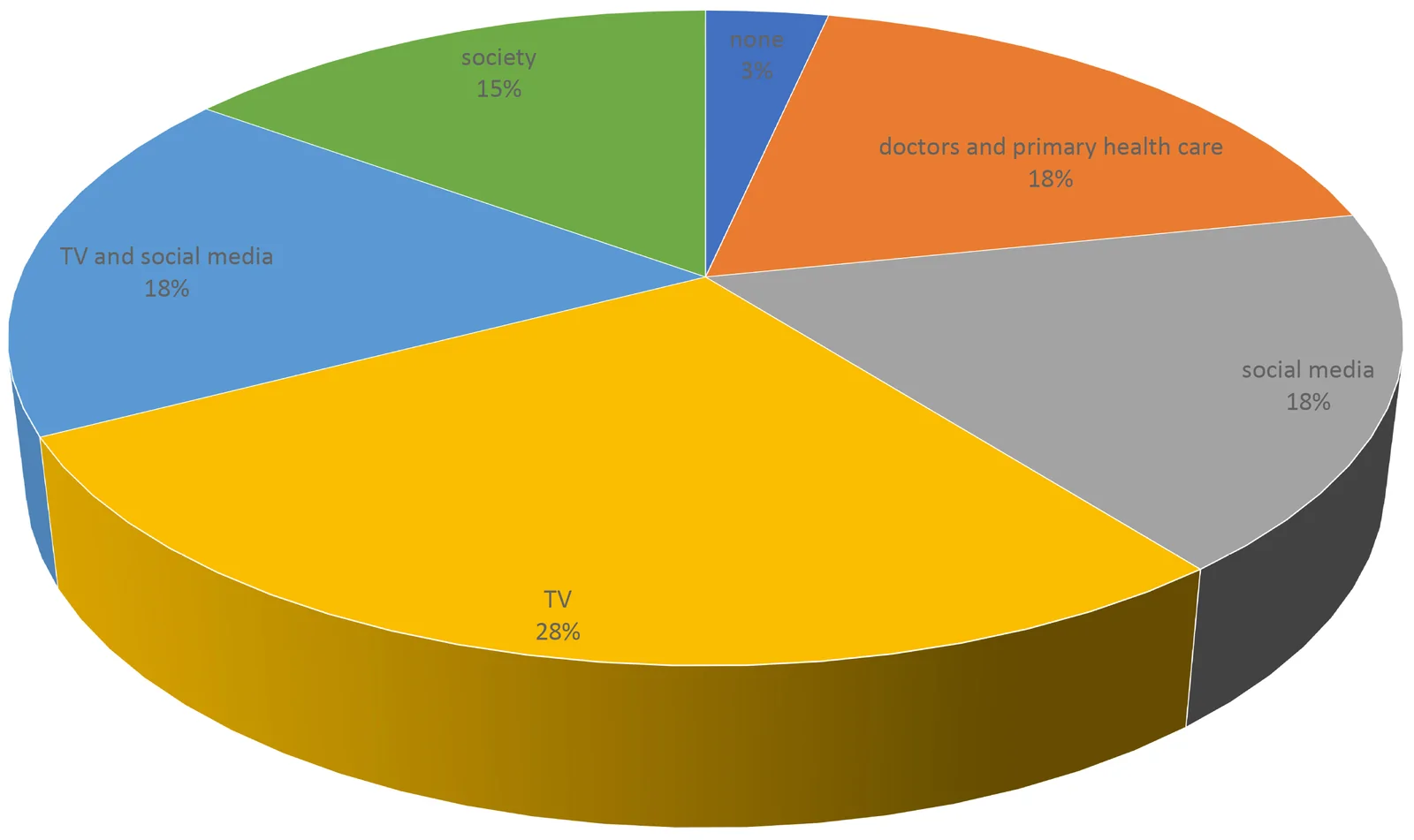
The main reason for not receiving the vaccine among patients receiving HD (n=183). HD, hemodialysis.

Among patients receiving HD who had not accepted the vaccination, the most frequently reported reason for refusal was fear of side effects (39.34%), followed by fear of infection (30.6%) and underlying medical conditions (13.11%). Other notable concerns included no perceived value in the vaccine (6.56%) and fear of reinfection (3.28%). Less common reasons were crowded vaccination sites (1.64%), misconceptions about unsuitability for dialysis (1.09%), workplace requirements (1.09%), waiting for the right time (1.09%), common cold (0.55%), and lack of vaccine availability at the preferred time (0.55%). A small number reported fear of thromboembolism (0.55%) (Fig 2).

### Factors Associated With Willingness to Receive Future Vaccine Doses

In total, 73.75% of patients receiving HD were willing to receive future doses of the COVID-19 vaccine. In the univariate analysis, participants with diabetes (model 2) were less likely to be willing to receive further doses (OR, 0.34; 95% CI, 0.14-0.83), as were those with hypertension (OR, 0.52; 95% CI, 0.34-0.78), cardiac disease (OR, 0.25; 95% CI, 0.09-0.71), and those who experienced hypertension during HD (OR, 0.51; 95% CI, 0.26-0.98). Participants with vaccinated relatives (model 3) were significantly associated with higher odds of willingness to receive another dose (OR, 2.38; 95% CI, 1.38-4.08).

In multivariable analysis, participants who did not have hypertension comorbidity (OR, 0.38; 95% CI, 0.18-0.79) or cardiac disease comorbidity (OR, 0.31; 95% CI, 0.10-0.97) (model 2), whose relatives had been vaccinated (OR, 33.8; 95% CI, 4.15-257.4), and whose relatives had not died from COVID-19 (OR, 0.06; 95% CI, 0.01-0.51) (model 3), were more likely to accept future vaccine doses.

### Source of Information About COVID-19 Vaccination

**Figure 3:**
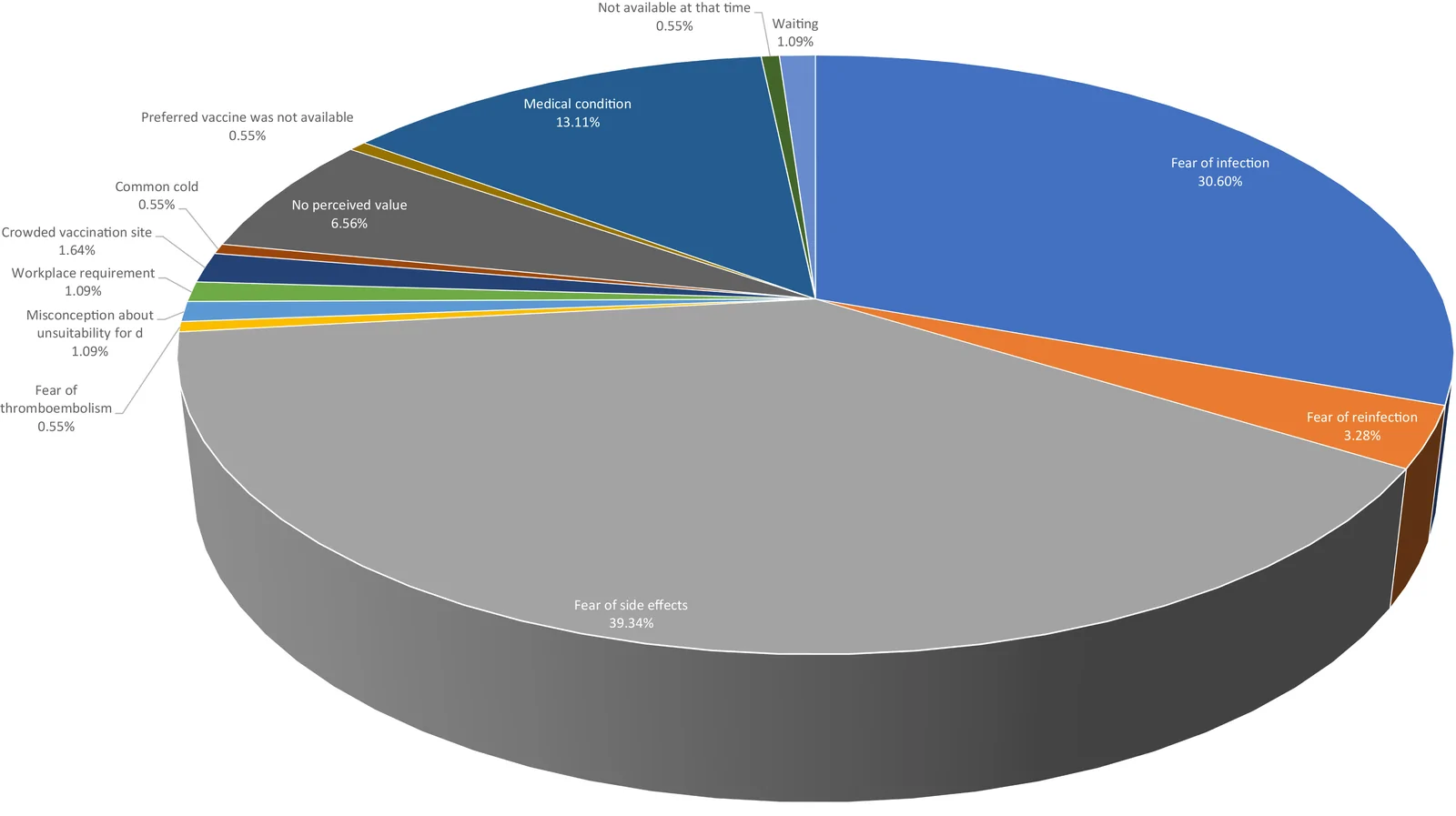
Main sources of COVID-19 vaccine information.

The majority of participants (97%) had access to information. In total, 63.27% reported mass media—particularly television and social media—as their main sources of information about COVID-19 vaccination, where television was reported by 27.89% of respondents, doctors and primary health care units by 18.36%, social media by 17.69%, and a combination of television and social media by 17.69%, whereas society accounted for 14.97%. A small proportion (3.4%) reported having no source of information (Fig 3).

### Reasons for Choosing a Particular COVID-19 Vaccine

**Figure 4:**
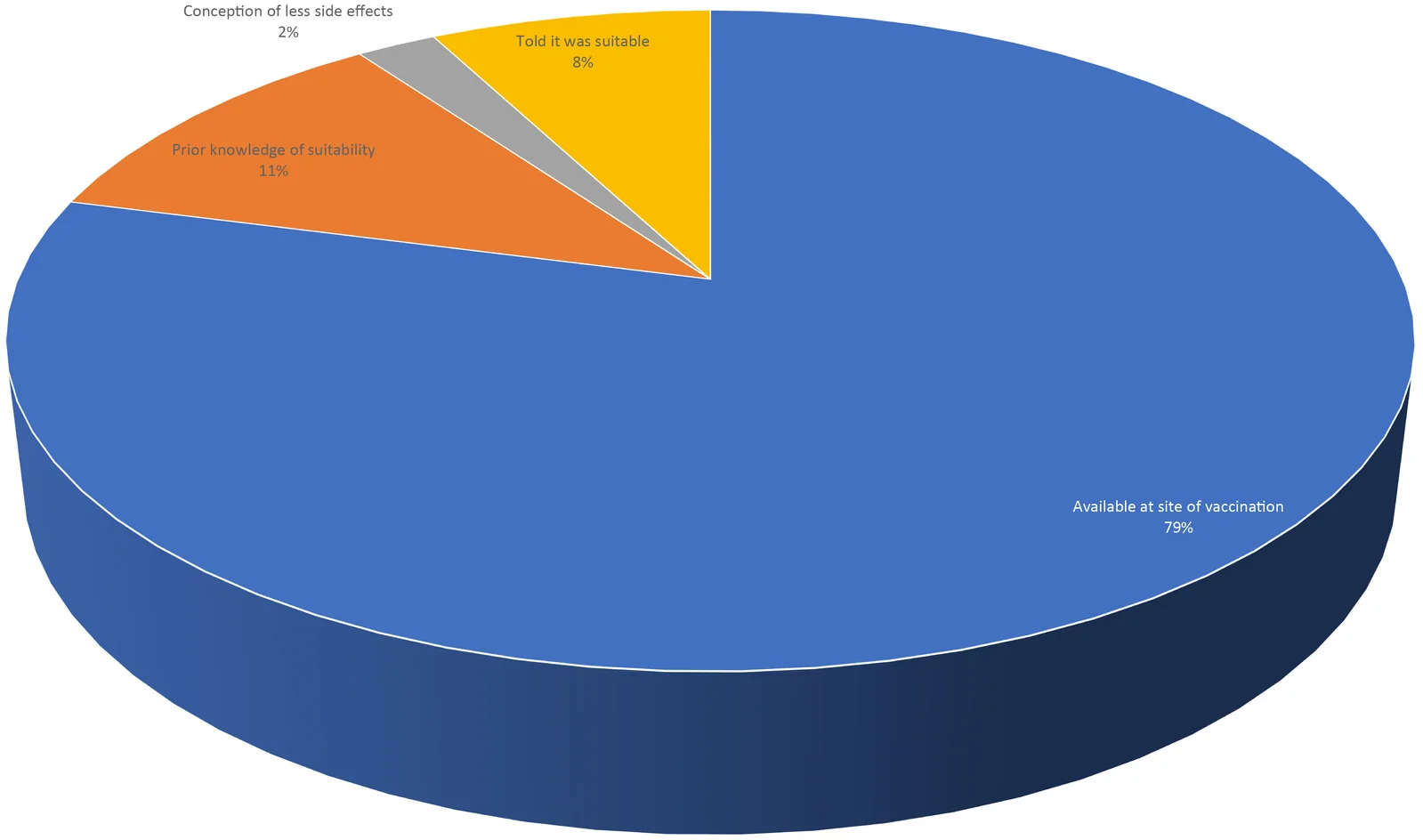
The main reason for choosing the vaccine among patients receiving HD (n=181). HD, hemodialysis.

The majority of participants (75.21%) reported that the availability of the vaccine at the site of vaccination was the primary reason for their choice. Other influencing factors included prior knowledge of the vaccine’s suitability (13.87%), being told it was suitable (7.56%), and the perception that it causes fewer side effects (3.36%; Fig 4).

### Predictors of Postvaccination Complications

The proportion that had postvaccination complications WAS 22.3%. In univariate analysis, married patients (model 1) were associated with higher odds of reporting complications (OR, 2.51; 95% CI, 1.63-3.86). Conversely, hypertension comorbidity (model 2) was associated with significantly lower odds of complications (OR, 0.28; 95% CI, 0.19-0.42); the presence of hypotension episodes during HD was associated with higher odds of complications (OR, 2.27; 95% CI, 1.34-3.85). COVID-19 reinfection (model 3) was associated with lower odds of complications (OR, 0.04; 95% CI, 0.01-0.16), whereas a history of relatives dying from COVID-19 (OR, 2.68; 95% CI, 1.57-4.60) and having vaccinated relatives (OR, 2.75; 95% CI, 1.80-4.19) was associated with higher odds of complications.

In multivariable analysis, married participants (OR, 5.83; 95% CI, 2.00-16.97) who were currently smokers (OR, 6.14; 95% CI, 1.40-26.88), who had undergone HD for a longer duration (OR, 1.01; 95% CI, 1.006-1.020) (model 1), and had a history of COVID-19 (model 3) (OR, 22.65; 95% CI, 5.24-97.91), were more likely to have postvaccination complications.

### Postvaccination Complications (Types)

**Figure 5:**
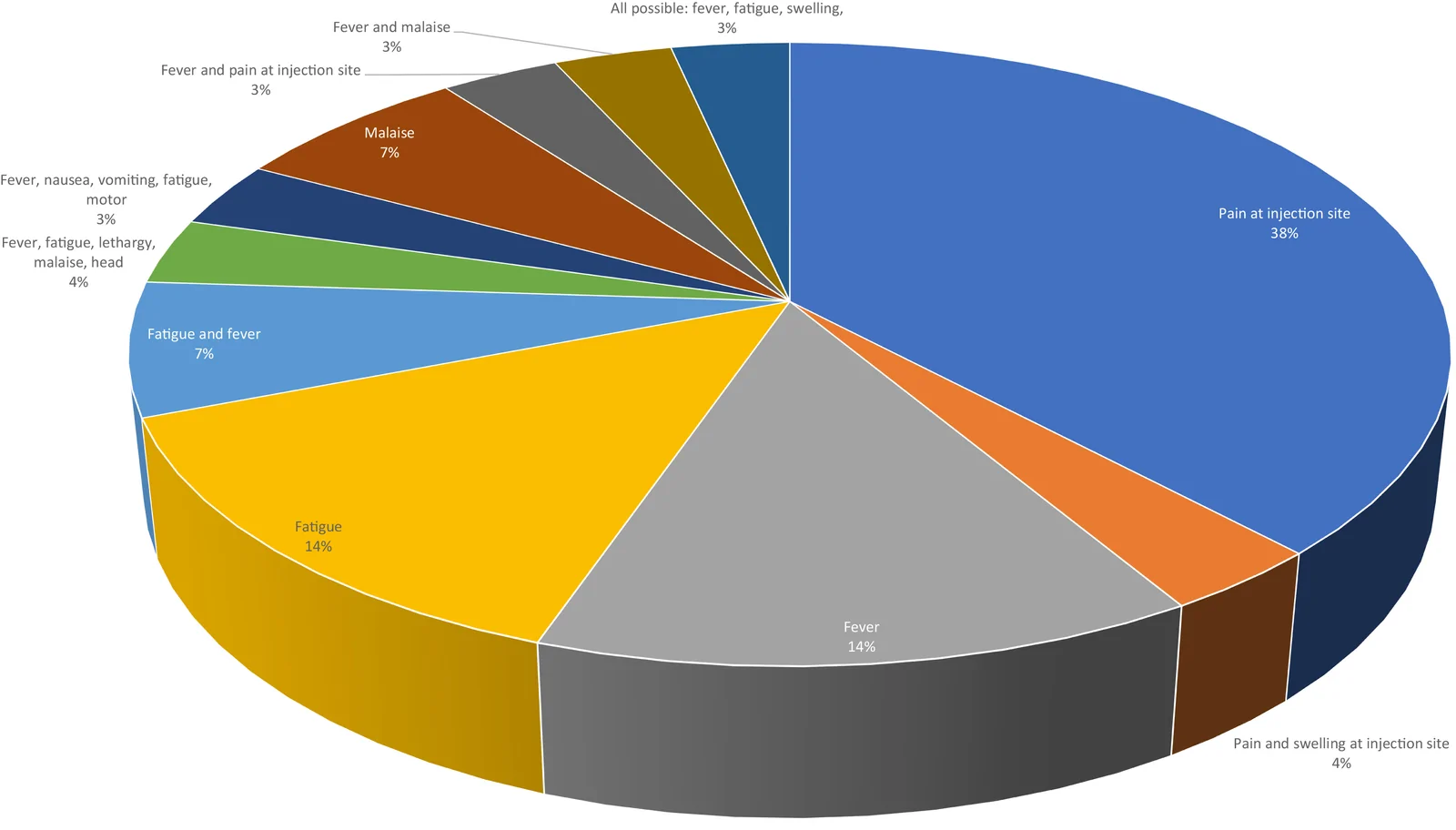
Postvaccination complications among patients receiving HD (n=29). HD, hemodialysis.

The majority of participants (55.8%) did not experience any complications following vaccination. The most frequently reported side effects among those who reported post-vaccination complications and reported the types of complications (n=29) were pain at the injection site (38%). Other reported complications included fever (14%) and fatigue (14%), whereas combined symptoms such as fatigue with fever (7%) and various combinations of fever, pain, swelling, and fatigue were reported collectively by 27% of participants (Fig 5).

## Discussion

This multicenter study assessed predictors of COVID-19 vaccine acceptance, willingness for future doses, COVID-19 before vaccination, and postvaccination complications in patients receiving HD in Egypt, Cameroon, and Kenya.

Patients receiving HD showed significantly lower vaccination rates (58.2%) than nondialysis controls (96.2%), consistent with known hesitancy among chronically ill populations.^10^ Our data showed that fear of side effects was the leading reason for vaccine refusal (39.34%), consistent with prior surveys among patients with kidney and other chronic diseases.^11^^,^^12^ Concerns about medical conditions (13.11%) and reinfection (3.28%) were common, reflecting perceived vulnerability to adverse events, highlighting the need for targeted education from trusted providers.^13^^,^^14^

The 96.2% vaccine acceptance among controls exceeds reported national estimates (eg, ∼42.6% in Egypt; < 10% full vaccination coverage in Cameroon and Kenya),^15^^,^^16^ likely reflecting selection bias because controls were relatives of patients receiving HD with potentially greater health awareness.

These discrepancies may reflect selection bias (relatives of patients may be more health-aware) and social desirability in reporting rather than true general population acceptance, because controls were relatives of patients receiving HD and may have greater health awareness or access to healthcare resources than the general population.

Despite being immunocompromised, patients receiving HD reported fewer postvaccination complications (22.3%) than controls (44.3%). This paradox could be because those who agreed to be vaccinated earlier were in better general condition; it may also reflect underreporting because of low health literacy, different symptom thresholds, vaccine type, immune hypo-responsiveness, or diminished response to the vaccine in this vulnerable group.^17^ Encouragingly, most participants (55.8%) had no complications. Among those who did, injection site pain (42%), fever (14%), and fatigue (14%) were most common, consistent with side effects seen in the general population.^18^, ^19^, ^20^ Previous studies also reported mild, self-limiting side effects after COVID-19 vaccination, with no rise in serious adverse events among patients receiving HD.^21^^,^^22^ These findings help alleviate fear and misinformation. The favorable safety profile should be highlighted in public messaging to boost vaccine confidence among patients receiving dialysis.

Older age, marital status, dialysis duration, prior COVID-19, and vaccinated relatives were significant predictors of vaccine uptake. In contrast, hypertension and reinfection were associated with lower odds. The positive association with age aligns with evidence that older adults perceive higher risk. Marital status likely reflects the role of social support in health decisions, as supported by the literature on social determinants of behavior.^14^^,^^23^, ^24^, ^25^, ^26^, ^27^ The association between hypertension and reduced vaccine acceptance was particularly notable and warrants further investigation, as hypertensive patients represent a high-risk group that would benefit most from vaccination.

Female sex was negatively associated with vaccination in unadjusted analysis, consistent with global trends.^28^ Dialysis-related complications were also inversely associated, possibly reflecting concerns about vaccine safety among patients already burdened by chronic complications.^29^

Notably, individuals with relatives who received the COVID-19 vaccine were nearly three times more likely to be vaccinated. This strong association highlights the impact of family and peer influence on vaccine acceptance, consistent with social network theory and vaccine uptake studies.^30^

Demographic and clinical factors had limited impact on willingness for future doses, but social exposure was critical. Having vaccinated relatives was the strongest positive predictor, whereas diabetes, hypertension, cardiac disease, reinfection, hypertension during HD, and relatives’ COVID-19 deaths were linked to reduced willingness. These findings highlighted how firsthand and social experiences shape health decisions, consistent with prior research.^31^^,^^32^ This suggests that family influence may be even more critical for ongoing vaccine acceptance than initial uptake.

The presence of vaccinated relatives was consistently linked with both uptake and willingness for future doses, highlighting the role of social influence.^33^^,^^34^

Among all evaluated predictors, anemia emerged as the strongest predictor of COVID-19 among patients receiving HD; this may reflect impaired immunity or nutritional deficiencies that compromise infection resistance. Prior studies have documented a link between anemia and increased susceptibility to respiratory infections, including COVID-19, which is plausible given the compromised immune status associated with anemia and immunosenescence. This suggests that addressing modifiable clinical factors like anemia could be a valuable target for reducing infection risk in dialysis populations.^35^^,^^36^

Although anemia was included as a predictor, it is recognized that both the presence of anemia and its underlying causes may influence vaccine acceptance. We acknowledge that causality cannot be determined in this cross-sectional study.

Postvaccination complications were significantly more frequently reported among married individuals, current smokers, and those with a history of COVID-19. The association with marital status may reflect better symptom recognition, reporting behavior, or healthcare access rather than true biological differences.^37^ The strong association between prior COVID-19 and postvaccination complications was a robust finding that could suggest an interaction between prior immune priming and vaccine-induced responses. However, this finding must be interpreted with caution, given the observational nature of the study and the potential for recall bias. Intriguingly, patients with dialysis-related complications were more likely to report vaccine side effects. This positive association may reflect heightened health awareness, increased interaction with healthcare providers, or a lower threshold for symptom recognition and reporting. Conversely, those who reported COVID-19 reinfection had lower odds of complications.

Moreover, mass media (television and social platforms) emerged as the primary source of vaccine information for 63.27% of participants. This aligns with global trends observed during the pandemic, where mass media served as a key channel for disseminating public health messages.^38^^,^^39^ However, social media, although accessible, has also been linked to the spread of misinformation, which can negatively impact vaccine confidence^40^—such as concerns about vaccines causing thromboembolism or being unsuitable for dialysis.^39^^,^^41^ The relatively lower reliance of patients receiving HD from health care professionals (18.36%) as a source of vaccine information is concerning. Previous studies have emphasized the importance of provider recommendation in improving vaccine uptake in vulnerable populations.^42^^,^^43^

Availability was the predominant factor influencing participants’ choice of vaccine (75.21%), underscoring the logistical nature of decision-making in resource-constrained or high-demand settings. Although only a minority based their choice on prior knowledge of suitability (13.87%) or perceived fewer side effects (3.36%), this reflects a passive acceptance rather than informed consent. This pattern suggests that while vaccine accessibility is crucial, efforts must also focus on improving health literacy to ensure that individuals can make well-informed choices about their care.^30^

Strengths of our study include its large, multicenter design across three different countries, enhancing generalizability and external validity. The inclusion of nondialysis controls provided the reference for assessing vaccine hesitancy in a high-risk group. A robust sample size and comprehensive questionnaire covering socio-demographic, clinical, and behavioral factors strengthened the analysis. However, this study has several limitations. First, the unequal distribution of participants—particularly the higher number of Egyptian patients compared with those from Cameroon and Kenya—may have influenced the observed prevalence of vaccine refusal across countries. Selection bias is also possible because not all cohorts were comparable, and the sample sizes of patients with kidney disorders (n=765) and nondialysis controls (n=196) were disproportionate.

Second, important clinical indicators related to end-stage renal disease—such as fluid overload, electrolyte abnormalities, dietary adherence, and mineral and bone disorder—were not assessed, limiting the ability to explore their association with vaccination outcomes. Temporal effects were not evaluated; thus, potential changes in vaccine acceptance over time could not be determined. Furthermore, the diversity of vaccine types and the absence of detailed information on vaccine selection limit interpretation of the findings.

Third, the cross-sectional design restricts causal inference, and reliance on self-reported data introduces potential recall and reporting bias. Recruitment from healthcare facilities may have excluded marginalized individuals with limited access to care, reducing the representativeness of the sample. Missing clinical data could have reduced analytic power. Finally, the absence of qualitative data prevents deeper exploration of cultural, social, and psychological factors that may influence vaccine hesitancy.

Fourth, a limitation of the study is that non-dialysis controls were not matched to the HD patients in number or demographic characteristics. Differences in age, sex, or health status between groups may have influenced responses, although multivariable analyses were performed to adjust for potential confounders. Additionally, some socio-demographic variables such as marital status were included based on prior literature,^24^ though their direct effect on vaccine uptake may be limited.

Fifth, some COVID-19 cases may have gone undetected, particularly asymptomatic cases, because testing was performed according to standard care at each center and not all participants underwent routine polymerase chain reaction screening.

Finally, the unequal representation across countries may limit generalizability, although all participants meeting inclusion criteria were enrolled.

COVID-19 vaccine hesitancy among patients receiving HD is driven by a complex mix of demographic, clinical, and contextual factors. Addressing this requires targeted public health strategies, including personalized education by trusted providers, improved vaccine access at dialysis centers, and combating misinformation—particularly for patients with hypertension and cardiac comorbidities. Future research should focus on vaccine immunogenicity, evaluate interventions to enhance uptake, and explore cultural differences influencing vaccination behaviors across different regions.

## Supplementary Material

Supplementary File (PDF) Table S1.

Supplementary File (PDF) Item S1.
