# Supplementary material for: COVID-19 Vaccine Knowledge, Practice, and Attitudes Among Hemodialysis Patients in Egypt, Kenya, and Cameroon: A Multicenter Study
Source: Kidney Med. 2026 Jul 14;8(9):101465. doi: 10.1016/j.xkme.2026.101465 (PMC13524677; doi:10.1016/j.xkme.2026.101465)
Supplement: Supplementary file 1 [file mmc1.pdf]

Figure S1. Causes of kidney failure among patients on SD.

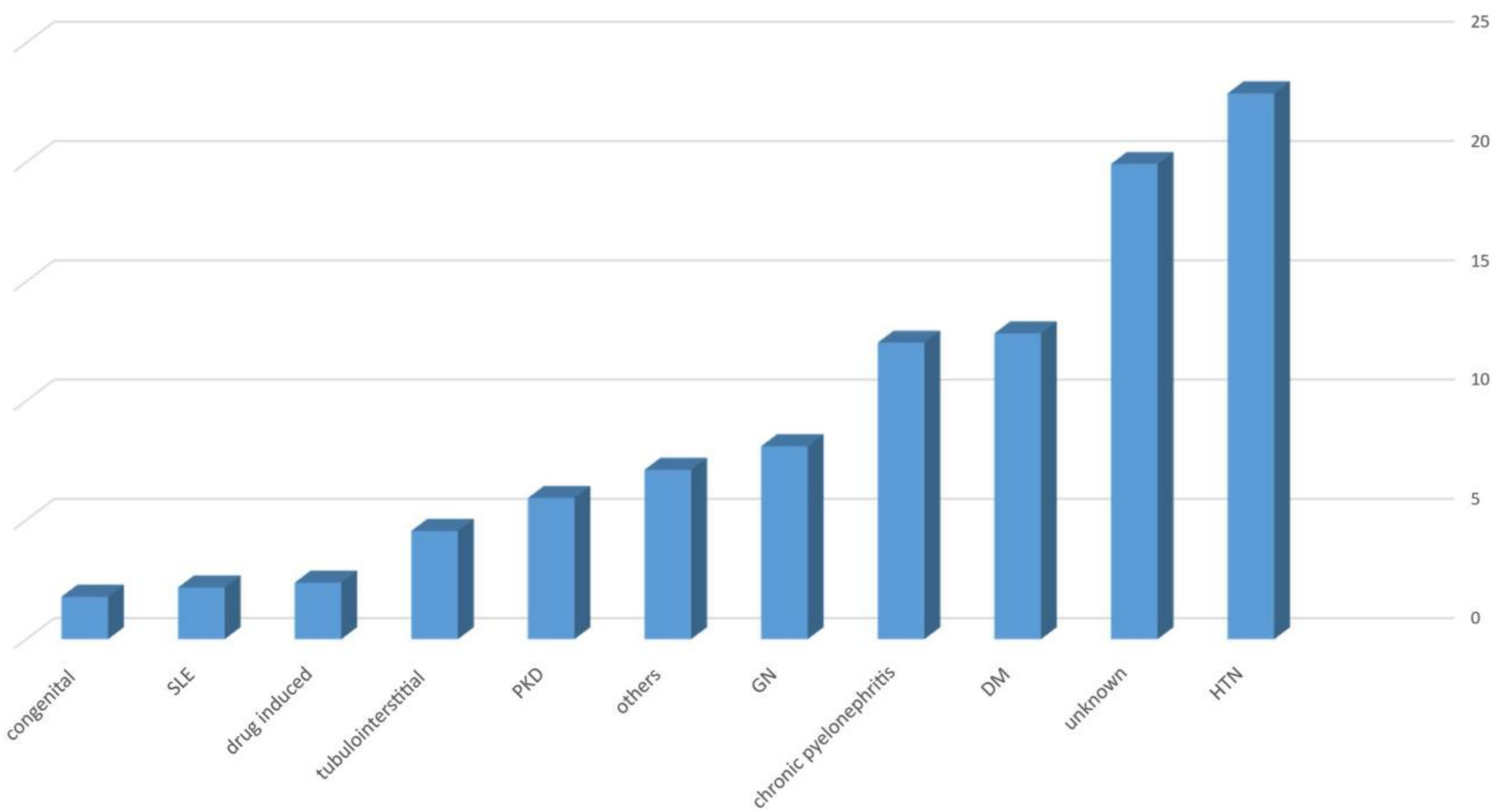

Abbreviations: SLE: Systemic Lupus Erythematosus; PKD: Polycystic Kidney Disease; GN: Glomerulonephritis; DM: Diabetes Mellitus; HTN: Hypertension
