# Supplementary material for: COVID-19 Vaccine Knowledge, Practice, and Attitudes Among Hemodialysis Patients in Egypt, Kenya, and Cameroon: A Multicenter Study
Source: Kidney Med. 2026 Jul 14;8(9):101465. doi: 10.1016/j.xkme.2026.101465 (PMC13524677; doi:10.1016/j.xkme.2026.101465)
Supplement: Supplementary file 2 [file mmc2.pdf]

Table S1. Baseline Characteristics of Patients Receiving Hemodialysis, Stratified by Country

Factor	Level	Egypt	Cameron (Yaounde)	Kenya (Nairobi)
N		636	100	29
Age, mean (SD)		50.0 (15.3)	48.3 (14.7)	55.9(11.9)
Age category, n (%)	<60	426 (69.2%)	77 (77.0%)	19 (70.4%)
	≥60	190 (30.8%)	23 (23.0%)	8 (29.6%)
Gender, n (%)	Male	348 (54.8%)	66 (66.0%)	20 (69.0%)
	Female	287 (45.2%)	34 (34.0%)	9 (31.0%)
Hemodialysis Duration (if any), mean (SD)		54.2 (63.5)	28.6 (29.3)	61.7 (55.6)
HD duration category, n (%)	<2 years	197 (44.9%)	65 (65.0%)	9 (34.6%)
	2-5 years	135 (22.4%)	23 (23.0%)	4 (15.4%)
	>5 years	344 (32.7%)	12 (12.0%)	16 (50.0%)
Educational Level, n (%)	None	75 (39.5%)	0 (0.0%)	0 (0.0%)
	Low	80 (42.1%)	18 (18.0%)	3 (12.5%)
	High	35 (18.4%)	82 (82.0%)	21 (87.5%)
Job Categories, n (%)	white-collar jobs	43 (21.4%)	81 (81.0%)	23 (85.2%)
	Retired	22 (10.9%)	0 (0.0%)	3 (11.1%)
	Manual / labor jobs	38 (18.9%)	19 (19.0%)	1 (3.7%)
	Not working	98 (48.8%)	0 (0.0%)	0 (0.0%)
Working, n (%)		516 (81.1%)	100 (100.0%)	26 (89.7%)
Smoking Status, n (%)	Current	99 (18.0%)	0 (0.0%)	1 (3.4%)
	Previous	77 (14.0%)	1 (1.0%)	9 (31.0%)
	Never	374 (68.0%)	99 (99.0%)	19 (65.5%)
Anemia, n (%)		110 (17.3%)	55 (55.0%)	0 (0.0%)
Diabetes, n (%)		26 (4.1%)	14 (14.0%)	0 (100.0%)
Hypertension During HD, n (%)	Yes	56 (12.8%)	0 (0.0%)	0 (0.0%)
Hypotension During HD, n (%)	Yes	76 (17.4%)	2 (2.0%)	0 (0.0%)

Country-level data are presented descriptively due to substantial imbalance in sample sizes and differences in dialysis practices acr oss participating centers.
