# Supplementary material for: COVID-19 Vaccine Knowledge, Practice, and Attitudes Among Hemodialysis Patients in Egypt, Kenya, and Cameroon: A Multicenter Study
Source: Kidney Med. 2026 Jul 14;8(9):101465. doi: 10.1016/j.xkme.2026.101465 (PMC13524677; doi:10.1016/j.xkme.2026.101465)
Supplement: Supplementary file 3 [file mmc3.pdf]

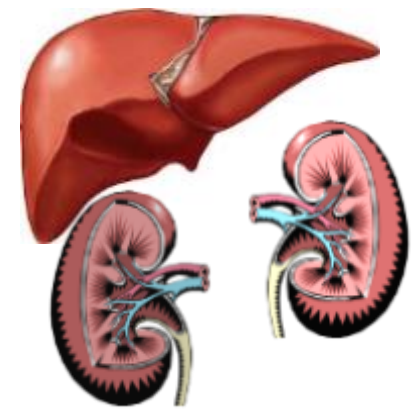

COVID-19 vaccination survey among patients on MHD

Type of participant :			***Patients on MHD***			***HDx relative***	
Name of hospital:							
Demography:				***Investigations:***			
Name :				Hb%	HCT	Plt	
ID:				WBCs	LYM	*Ferritin:*	
Phone:				*CRP*	*BUN b*	*BUN a*	
Age:	*Gender :*			*URR*	*Creat*	*D Dimer*	
Smoking:	*current*	*previous*	*Never*	*LDH*	*albumin*	*Ca*	
Married :	*address:*			*Phosphrus*	*iron*	*TIBC*	
				PTH	*Vit D*	*Alk phos*	
Level of education:				*U acid:*	*AST*	*ALT*	
Job:	*HDx Access:*			*RBS*	*T bil*	*D bil*	
Cause of RF:				Virology			
Date of start HDx:				***Vaccination:***			
Session duration:	*Frequency:*			*yes*	*No*	*Why?*	
Wt b:	*Wt a:*	*Hight:*					
Any complication during HDx	*Type of complication:*			*Type of vaccine*		*Date*	
Comorbidities :				*Why this vaccine?*			
DM:	*HTN:*	*Cardiac:*		Doses:	1st	2nd	3rd
Lung:	*Liver:*	*Malignancy:*		Will you receive the other dose:			
Pregnancy:	*Chemo:*	*Immunosup:*		Your source of knowledge about vaccine			
CKD:	*Previous Tx:*	*Oral anticoag:*					
History of previous COVID-19:				Do you believe its important to receive it?			
Yes:	***No:***	***Contact with case:***		***IgG before***		***IgM before***	
Frequency:	***Date :***			***Post vaccination side effects:***			
Diagnosis	***CBC:***	***CT:***	***PCR:***	*Fever*	*Fatigue*	*Lethargy*	
Received ttt:	***Type of isolation:***			*Malese*	*Pain at injection site*		
Severity:	***Need MV:***			*Swelling at injection site*		*stiffness*	
Symptoms:				*Itching or redness*		*Skin rash*	
Fever:	*Cough:*	*Sore throat:*		*Chills*	*Allergic reaction*		
Dyspnea:	*Fatigue:*	*Myalgia:*		*Joint pain*	*Headache*	*Blurring vision*	
Diarrhea:	*Vomiting:*	*Abd pain:*		*Dizziness*	*Flu like symptoms*		
Chest pain:	*Taste loss:*	*Anosmia:*		*Tingling*	*Motor weakness*		
Oliguria:	*Thrombotic events:*			*Nausea*	*Vomiting*	*Palpitation*	
Drowsiness:	*Skin eruption*			*SOB*	*Sleep disturbance*		
Others:				*Sweating*	*Hotness*	*Coldness*	
Post COVID complications:				*Leg swelling*	*Calf pain*	*Chest pain*	

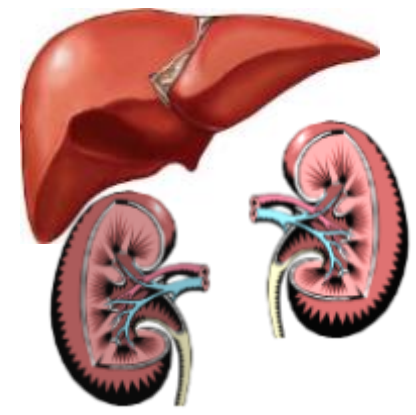

LN swelling	*Easy bruising*	***Drug history:***	
Thrombotic events	*Others:*	*hydroxychloroquine*	*Ivermectin*
Post vaccination COVID-19		*Zinc*	*Vit c*
Symptoms:		*Insulin*	*metformine*
Severity:		*Oral antidiabetic*	*Clexan*
IgG after	*IgM after*	*Paracetamole*	*Aspirin*
Other vaccination		*NSAID*	*Favipiravir*
Did you received :		*Actimra*	*Dexamethasone*
Influnza vaccine?		*Oral steroids*	*Inhaled steroids*
HBV vaccine?		*Inhaled B agonist*	*Lactoferrin*
Did you respond to other presidential health initiatives?		*Acetylcysteine*	*Oxygen*
Relatives		*Vit D*	*ACEI*
Any relative had COVID-19?		*Anti HTN drugs*	
Degree of relative			
Any relative died from COVID-19?			
Degree of relative			
Any relative received the vaccine:			
Degree of relative?			
His phone number			

Doctor signature:
